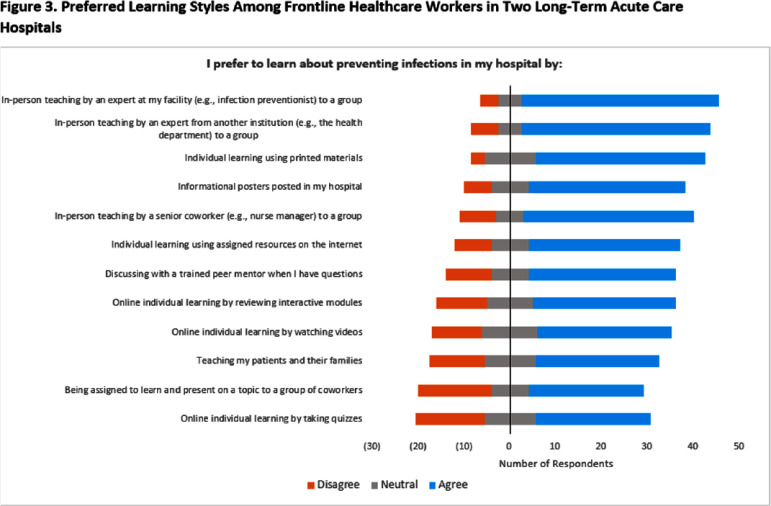# Barriers to Candida auris Prevention Among Frontline Healthcare Workers in Long-Term Acute Care Hospitals

**DOI:** 10.1017/ash.2025.226

**Published:** 2025-09-24

**Authors:** Sarah Sansom, McKenzi King, Christine Lucky, Lisa Duffner, Lahari Thotapalli, Laura Rusie, Rachel Medernach, Mary Carl Froilan, Nidhi Undevia, Mary Hayden

**Affiliations:** 1 Rush University Medical Center; 2 RML Specialty Hospital

## Abstract

**Background:** Gaps in knowledge and compliance regarding control of Candida auris contribute to ongoing spread in healthcare facilities, but few studies have evaluated barriers to effective prevention measures. **Methods:** We assessed the knowledge, attitudes, beliefs, and practices for control of C. auris among frontline healthcare workers at 2 long-term acute care hospitals (LTACH) with high admission prevalence of C. auris in the Chicago, Illinois region. Surveys included 92 questions including the following subjects: participant demographics; awareness of multidrug-resistant organisms [MDRO]; attitudes and beliefs regarding C. auris; motivation for using and compliance with personal protective equipment [PPE]; knowledge of C. auris prevention measures; perceived barriers and facilitators of C. auris prevention; sources of education and training; and preferred learning styles. Responses were measured on a 5-point Likert scale. Anonymous online surveys were administered during the one-month study period Recruitment efforts included posters, fliers, email, and in-person rounds with the LTACH infection preventionist. Participants were eligible to win a gift card upon survey completion. Only complete surveys were analyzed. **Results:** Fifty-three surveys were completed (estimated 12% response rate across all facility staff) with respondents identifying as 92% female, 43% nurses, and 60% with >10 years of experience in their current role. Participants were familiar with commonly identified MDROs (i.e., Clostridoides difficile [98%], methicillin-resistant Staphylococcus aureus [88%]) but were less aware of extended-spectrum β-lactam-resistant (68%) and carbapenem-resistant pathogens (56-79%) (Figure 1). Participants felt that their actions helped prevent C. auris spread (85%) but were less confident when asked whether C. auris is a problem at their own hospital (53%). Participants were able to successfully identify most prevention strategies for C. auris (≥72% correct for all measures). The highest ranked barriers to C. auris prevention were understaffing (63%) and inadequate training (51%) (Figure 2). Information regarding C. auris was most commonly obtained through training provided within their hospital (62%) and from coworkers (45%). Participants responded that they prefer to learn about infection prevention through in-person teaching by experts at their facility (83%) or from another institution (79%), although self-learning styles were also popular (67-73%) (Figure 3). **Conclusion:** We identified perceived barriers to effective C. auris prevention among frontline healthcare workers in 2 LTACHs. While staff members successfully identified most prevention strategies for C. auris, they may benefit from enhanced education and training programs that support multiple learning styles.

**Figure:**
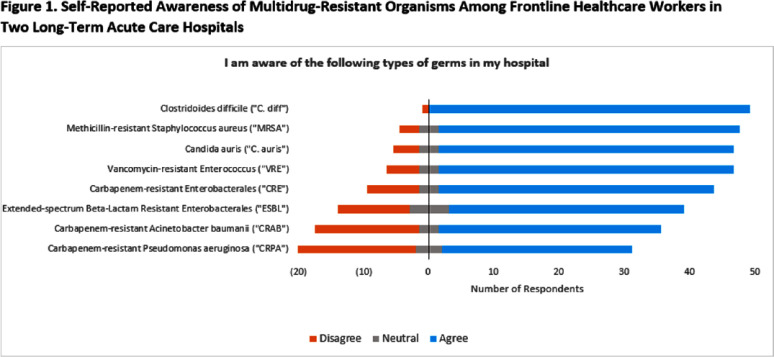

**Figure:**
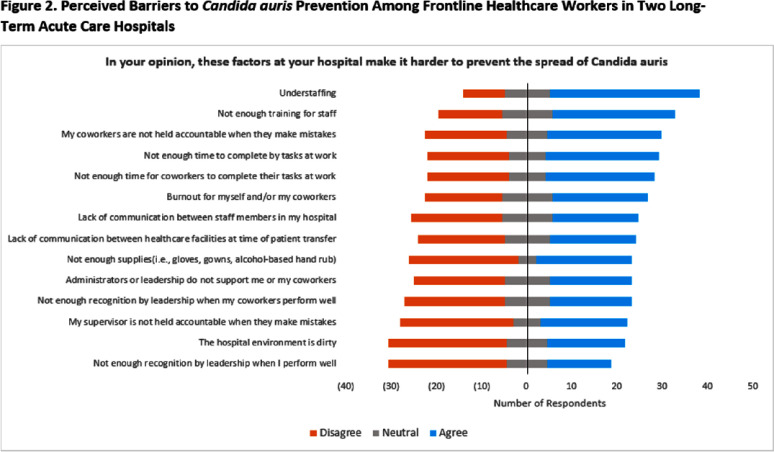

**Figure:**